# Distinct co-evolution patterns of genes associated to DNA polymerase III DnaE and PolC

**DOI:** 10.1186/1471-2164-13-69

**Published:** 2012-02-14

**Authors:** Stefan Engelen, David Vallenet, Claudine Médigue, Antoine Danchin

**Affiliations:** 1 AMAbiotics SAS, Bâtiment G1, 2 rue Gaston Crémieux, 91000 Evry, France; 2 CEA, DSV, IG, Genoscope & CNRS-UMR8030, Laboratoire d'Analyse Bioinformatiques en Génomique et Métabolisme (LABGeM), 2 rue Gaston Crémieux, 91057 Evry, France; 3 Department of Biochemistry, Faculty of Medicine, Hong Kong University, 21 Sassoon Road, Pokfulam, Hong Kong

**Keywords:** replication, degradosome, LUCA, phylogenetic profiling, nanoRNase

## Abstract

**Background:**

Bacterial genomes displaying a strong bias between the leading and the lagging strand of DNA replication encode two DNA polymerases III, DnaE and PolC, rather than a single one. Replication is a highly unsymmetrical process, and the presence of two polymerases is therefore not unexpected. Using comparative genomics, we explored whether other processes have evolved in parallel with each polymerase.

**Results:**

Extending previous in silico heuristics for the analysis of gene co-evolution, we analyzed the function of genes clustering with *dnaE *and *polC*. Clusters were highly informative. DnaE co-evolves with the ribosome, the transcription machinery, the core of intermediary metabolism enzymes. It is also connected to the energy-saving enzyme necessary for RNA degradation, polynucleotide phosphorylase. Most of the proteins of this co-evolving set belong to the persistent set in bacterial proteomes, that is fairly ubiquitously distributed. In contrast, PolC co-evolves with RNA degradation enzymes that are present only in the A+T-rich Firmicutes clade, suggesting at least two origins for the degradosome.

**Conclusion:**

DNA replication involves two machineries, DnaE and PolC. DnaE co-evolves with the core functions of bacterial life. In contrast PolC co-evolves with a set of RNA degradation enzymes that does not derive from the degradosome identified in gamma-Proteobacteria. This suggests that at least two independent RNA degradation pathways existed in the progenote community at the end of the RNA genome world.

## Background

Future developments of Synthetic Biology require that patterns of gene organization in genomes are carefully taken into account [1]. Following the pioneering work of Sueoka [2], Lobry and co-workers identified a replication-linked bias in the nucleotide distribution in bacterial chromosomes. Subsequently, a bias in favor of genes transcribed in the same direction as that of the movement of the replication fork was observed in most bacterial genomes [3,4]. The bias was correlated with the presence in the genome of a single origin of replication. Taken together, these observations led to the construction of algorithms meant to identify in silico the origins of replication [5]. The cause of the bias has been a matter of speculation until it was observed that Firmicutes displayed the strongest bias [6,7] reaching 87% in organisms such as *Thermoanaerobacter tengcongensis *[8]. A first hypothesis proposed that the bias was favoring genes requiring high expression [9]. Yet, there was no correlation between gene expressivity and transcription from the leading strand. Indeed, many genes of the replications machinery are expressed at a low level, and they are transcribed from the leading strand [10]. The leading strand bias had therefore to be accounted for by physical constraints: transcription of key genes must avoid head-on collision with the replication machinery to prevent formation of truncated transcripts. The latter are known to be toxic for the cell, in particular when they code for polypeptides belonging to protein complexes (see [11] for a general description of the process).

A further observation noticed that organisms that were strongly biased in the leading vs lagging strand replication encoded two DNA polymerases III, DnaE and PolC, rather than a single one [12]. DnaE was originally identified in *Escherichia coli *[13] whereas PolC was identified in *Bacillus subtilis *[14]. Yet, in contrast with the situation in Eukarya where the presence of two polymerases is the norm, most bacterial species listed in genome reference databases code for only one DNA polymerase III. In *E. coli*, the same structural type of DnaE replicase acts on both the leading and lagging strand. Two identical replicase molecules are held together in a complex with the replicative helicase and subunits with priming activities, allowing two identical alpha catalytic subunits to assume different functions on the two strands of the replication fork [15]. In contrast, in *B. subtilis*, asymmetric DNA synthesis requires replicative DNA polymerases with two distinct structures, DnaE and PolC. In contrast to PolC, DnaE, which replicates the lagging strand, is devoid of 3' --> 5'-proofreading exonuclease activity and has a low processivity (1-75 nucleotides), requiring additional factors to fulfill its role in replication [16].

DnaE and PolC differ both in structure and activity [15]. This prompted us to explore whether their genes co-evolved with consistent groups of genes, allowing us to propose scenarios of the origins of the replication machineries. In particular the cell manages compartmentalization via the cell's envelope, appendages, but also nanomachines such as the ribosome, ATP synthase, the degradosome and many others [17]. We present here a phylogenetic profile analysis focused on the bacterial *dnaE *and *polC *genes and show that proteins co-evolving with PolC have distinct features, and may form a specific kind of degradosome. The consequences in terms of the origin of bacteria are discussed.

## Methods

To separate between the history of DnaE and that of PolC, we established a heuristics meant to identify genes that co-evolved in bacterial genomes. The approach is straightforward: we first identify orthologous genes by pairwise comparison to compute phylogenetic profiles; we subsequently compare them after statistical validation, taking into account the phylogenetic distances between organisms with co-occurring genes; finally, we combine phylogenetic profiling with other methods that take into account the genomic context.

### Pairwise gene comparison and phylogenetic profiles

Phylogenetic profiling uses binary vectors that, taking genes one by one, identify in which organisms an ortholog is present (resp. absent). To this aim, Tatusov introduced the notion of "occurrence vector" for groups of orthologous proteins [18]. Here we used the complete RefSeq NCBI database of bacterial genomes [19] comparing the cognate proteomes using BlastP (all genes of a proteome against all proteomes). We subsequently identified orthologs using bidirectional best hits (BBH) as described by Koonin and co-workers [20]. Next, we retained orthologs according to the distribution of a gene similarity scores, **s**, designed to take into account biological constraints other than orthology, using relevant thresholds (see equation 4 for definition of the thresholds).

Lacking an evolutionary model common to all genomes, we chose the simplest model: the similarity score we used is the direct convolution of identity (**i**) and coverage (**c**: length of the BlastP alignment divided by the length of the longest protein) of the BlastP hits:

(1) s=i*c

**Figure 1:**
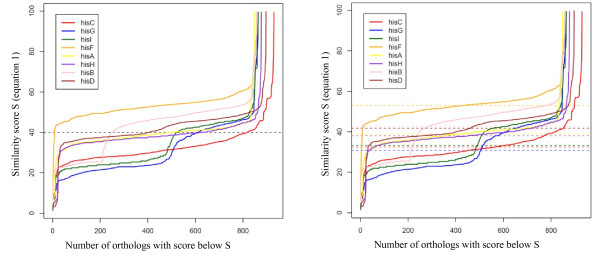
**Curves of sorted similarity score s (equation 1) for 8 genes of the *B. subtilis *histidine pathway**. The x axis represents the number of orthologs of the target gene having a **s **score below y. Left panel: The same threshold (dashed black line at 40) for all genes is used. Right panel: Different thresholds chosen according to the average of **s **are used for each gene.

We computed the values of **s **for each gene ortholog present in the target organism. As an example, Figure 1 (left panel) displays the behavior of sorted **s **for 8 genes of the *B. subtilis *histidine pathway. The x axis indicates the number of orthologs of the target gene having a **s **score below x. The curves displayed in Figure 1 show that if we used a common threshold (score of the y axis) for all genes, we would find inconsistent levels of orthology. For example, fixing the threshold at 40 (arbitrary units, dashed black line) for the gene *hisF *retained almost all *hisF *orthologs but only some *hisC *orthologs (selected orthologs have similarity scores above the threshold). Ths is because there is no common molecular evolutionary clock [21] for the genes in the pathway: *hisC *orthologs genes maintain their function despite a high rate of mutation, generating BlastP hits with lower similarity scores. This constrasts with the evolution of *hisF *orthologs. Had we used the same threshold for *hisC *and *hisF *genes we would not have found them to be correlated.

We therefore selected orthologous genes verifying the following formula:

(2) s>avg(s)

This allowed us to select orthologous genes with a similarity score **s **above the average of **s, avg(s) **(see Figure 1 right panel). Now, the number of orthologous genes selected for the genes *hisF *and *hisC *is almost the same. As a consequence, if these orthologs belong to the same organisms, the two genes will be found to be correlated, a functionally relevant observation.

Next, we included in the model the fact that orthologous genes must have a similar homology score because positive selection pressure tends to retain only a limited number of mutations among those that are constantly created. This implies that a high density of similarity score should correspond to families of organisms that keep the function of a gene with no alteration. To this aim, we first computed the distribution of **s **values, **d(s)**, for each ortholog of target genes.

**Figure 2:**
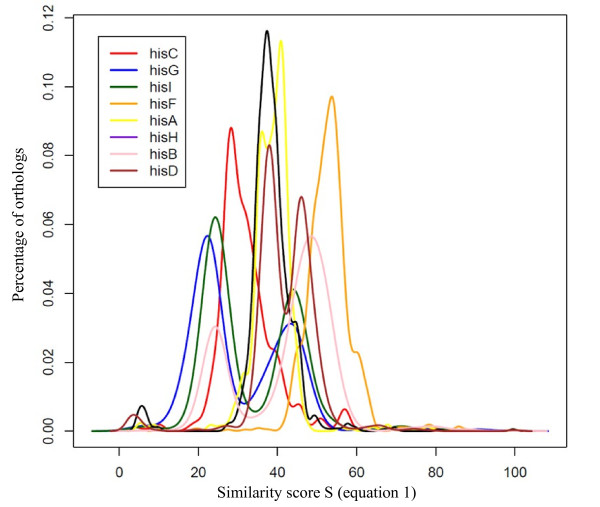
**Distribution d(s) of orthologs s values (equation 1) for the histidine pathway's genes of *B. subtilis***.

Figure 2 shows how **d(s) **varies for the histidine pathway's genes of *B. subtilis*. We subsequently retained the orthologs genes verifying the following formula:

(3) d(s)>avg(d(s))

Finally, we took into account the fact that proteins may keep their function while suffering different mutation rates (a different threshold for each gene) and that families of organisms that keep the function of a gene have similar homology scores because they undergo positive selection pressure (high densities of homology score). To this aim we selected orthologs with a similarity score **s **above the average and otherwise orthologs genes with a **s **density above the average, combining (2) and (3).

(4) s>avg(s)ORd(s)>avg(d(s))

**Figure 3:**
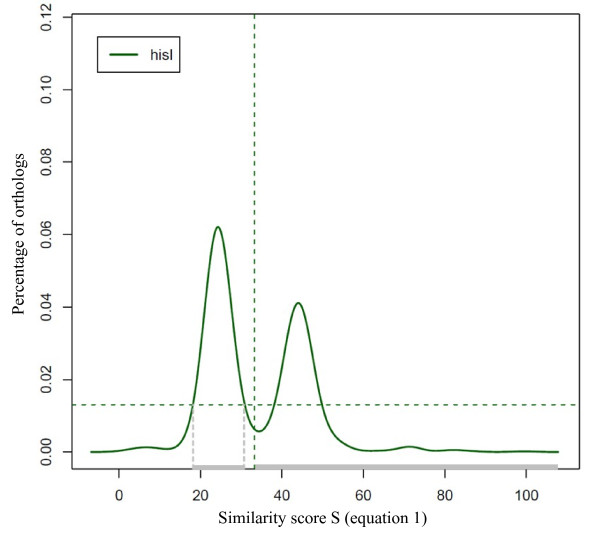
**Distribution d(s) of orthologs s values (equation 1) for gene *hisI *of *B. subtilis***. Orthologs of *hisI *which have their **s **values indicated in grey correspond to orthologs selected applying equation 4.

Figure 3 shows an example of this selection (selected orthologs genes have their **s **values indicated in grey) for *hisI *orthologs.

Relevant selection of orthologs has been computed for each gene of the target organism. This allowed the building up of binary vectors of presence/absence for these genes to explore the hypothesis that functionally linked genes have the same occurrence vectors. The next step was then to compare together these occurrence vectors to underscore functional relationships that group together genes of the target organism.

### Comparison of phylogenetic profiles

As previous authors, we assumed that in the course of evolution functionally-related genes tend to be gained or lost together. This results in a correlation of their occurrence vectors. The first exploration of this hypothesis compared co-occurrence profiles using Hamming's distance [22]. Subsequently, many different statistical approaches to compare phylogenetic profiles have been used, such as mutual information [23], Pearson correlation coefficient [24] and Fisher's test [25]. Here we used the phi coefficient to compare phylogenetic profiles of two genes X and Y. This measure is similar to the Pearson correlation coefficient. In fact, a Pearson correlation coefficient estimated for two binary variables will return the phi coefficient:

(5) $$\phi =\left(n_{11}*n_{00}-n_{10}*n_{01}\right)/{\left[\left(n_{11}+n_{10}\right)\left(n_{01}+n_{00}\right)\left(n_{10}+n_{00}\right)\left(n_{01}+n_{11}\right)\right]}^{1/2}$$

With n_11_, the number of organisms in which X and Y are present; n_00_, the number of organisms in which X and Y are absent; n_10_, the number of organisms in which X is absent and Y is present; n_01_, the number of organisms in which X is present and Y is absent. The formula is symmetric:

(6) ϕ(X,Y)=ϕ(Y,X)

### Measure of phylogenetic distances

The Pearson correlation, as other statistical methods, ignores that organisms are phylogenetically related and that the phylogenetic kinships may be biased. This must be taken into account, as the genome samples that have been sequenced is considerably biased, in terms of relative phylogenetic proximity. This may have a negative influence on our predictions. To reduce the effect of this phylogenetic skew, we modified the formula, taking into account the phylogenetic distance between organisms in which the genes co-occur. The idea was to give a larger weight to genes co-occurring in distant organisms than to those present/absent simultaneously in closely related organisms. As we do not have a detailed model that would account for the sampling bias in genome data, we used a plausible straightforward phenomenological measure of the proximity between two organisms, A and B, D(A,B):

(7) $$D\left(A,B\right)=1-\left(N\left(A,B\right)/max\left(N\left(A\right),N\left(B\right)\right)\right)$$

with N(A,B) the number of genes occurring in organisms A and B, N(A) the number of genes in organism A and N(B) the number of genes in organism B.

In the absence of a model describing genome evolution, and knowing that there is a considerable anthropomorphic bias in the choice of the organisms that have been sequenced we chose a somewhat arbitrary non-linear model to increase the weight of distant organisms. Our formula to measure the functional link between two genes X and Y is now:

(8) $$\phi (X,Y)*D{\left(A,B\right)}^{3}$$

with A and B the most distant organisms in which genes X and Y co-occur.

### Coupling with genomic context methods

Further biologically-relevant factors must be taken into account to construct a plausible heuristics. A great many methods use the genomic context of genes to predict functional links between proteins. For example, functional links are suggested by conservation of gene neighbourhood and gene order [26], gene fusion events [27], correlation of the genes' evolutionary rate [28] and correlation of genes' occurrence in organisms (phylogenetic profiles). In [23] an evaluation of the methods that emphasized these factors showed that conservation of the gene neighborhood and the gene order covered 45% of the functional interaction between genes of *Mycoplasma genitalium*. In the present work, the gene neighborhood was measured using the Syntonizer software (implemented in the MicroScope platform [29]), which is based on an exact graph-theoretical approach to measure synteny [30]. A factor K was used: K(X,Y) = 1 if the two genes X and Y are found at least once in synteny and 0.9 otherwise.

The final formula to compute the co-evolution score **C **between two genes X and Y is then:

(9) $$C(X,Y)=\phi (X,Y)*D{\left(A,B\right)}^{3}*K(X,Y)$$

with ϕ(X,Y) the correlation score between phylogenetic profiles of the genes X and Y, D(A,B) the distance between the most distant organisms A and B in which the genes X and Y co-occur and K(X,Y) a factor measuring the conservation of genes neighborhood.

This phylogenetic profile method (PhyloProfile) has been integrated in the MicroScope platform [29]. It is directly available in the gene editor and allows the users to compute dynamically co-evolution scores of the target gene against all genes of the organism of interest.

### Construction of clusters of co-evolution

Finally, following computation of the relevant phylogenetic profiles, we constructed co-evolution clusters. To this aim, we computed co-evolution scores, **C**, for all genes of *B. subtilis, E. coli *and *Acinetobacter baylyi*. If n is the number of genes of an organism, we obtained n*(n-1) scores. These scores were used to build up networks in which nodes correspond to genes and edges correspond to scores of co-evolution between genes. Applying a clustering method on these networks will allow construction of partitions of these genomes into clusters of co-evolving genes. Here we used as a clustering method the Markov Cluster algorithm (MCL; Van Dongen 2000; http://micans.org/mcl/) that is designed to cluster large numbers of relationships in a similarity space. The MCL algorithm is a fast and scalable unsupervised cluster algorithm for networks, based on simulation of flows in graphs. It has successfully been applied for clustering large sets of protein sequences [31,32]. In the present work, clusters corresponding to functionally relevant processes, such as metabolic pathways (histidine biosynthesis...) or global functions (degradosome ...) were obtained, depending on the threshold used in the clustering procedure.

As a validation of our approach we verified that molybdopterin biosynthesis and use have disappeared in a concerted way, as observed in *Pseudoalteromonas haloplanktis *[33].

## Results

The contrasted replication-associated gene orientation bias uncovered between most bacterial clades and Firmicutes prompted us to explore the underlying phylogenetic constraints that support this discrepancy. To this aim we meant to uncover the functions that co-evolved with either DnaE or PolC.

**Figure 4:**
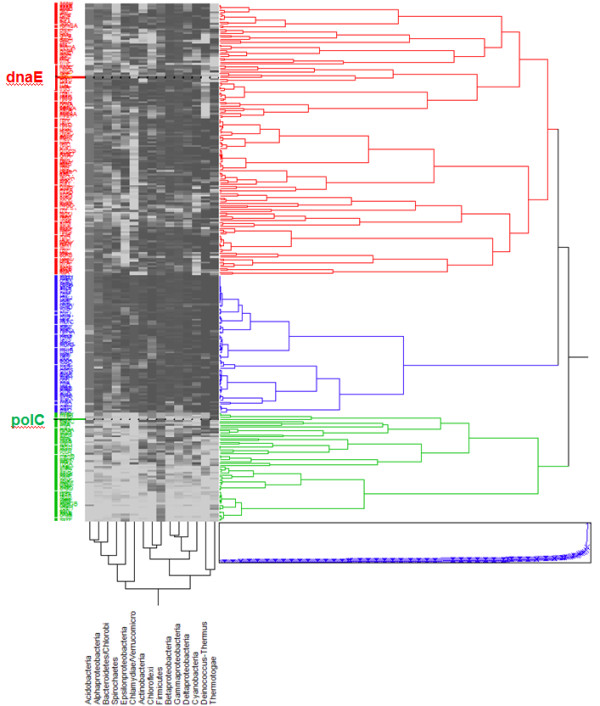
**Hierarchical clustering of occurrence proportion vectors of *B. subtilis *essential genes in clades of the domain Bacteria**. Squares show the proportion of a phylum (ascending from white to black) having an occurrence of a gene. The dendogram depicts distances between the proportion vectors. This dendogram separates *B. subtilis *essential genes into three clusters. The first one (red) contains *dnaE *and corresponds to genes occurring in the majority of bacterial clades. The second one (blue) contains *dnaA *and spans the whole domain of bacteria. The last one (green) contains *polC *and is composed of genes essentially specific to Firmicutes. The computing and visualization of the clusters was performed using the JMP^® ^software (SAS Institute, Cary, NC).

To get a first crude view of the processes underlying this replication-associated bias we used the JMP^® ^software (SAS Institute, Cary, NC) to compute a hierarchical clustering of the *B. subtilis *essential genes [34] according to their occurrence proportion in different Bacteria clades (Figure 4). Three main clusters were obtained. Unexpectedly, they were all related to DNA replication. This substantiated the conjecture that the way replication is organized was indeed at the core of some important functional variation specific to bacteria forming a given clade.

We observed the following, from top to bottom in Figure 4: A first cluster corresponded to genes present in almost all bacterial clades. This cluster contains *dnaE*, which, however, seems to be absent from the Deinococcus-Thermus and Chlamydia-Verrucomicrobia clades. It also comprises the bulk of the translation machinery (ribosome protein genes), including a limited number of tRNA synthetases, RNA polymerase genes, core iron-sulfur metabolism, and the core genes required to synthesize an envelope (Additional file 1). The second cluster (in blue) corresponded to genes spanning the whole domain Bacteria. It comprizes most genes of tRNA synthetases and the remaining set of ribosomal protein genes. Remarkably it clusters with *dnaA *as well as the gene for DNA primase. Finally, the third cluster corresponded to genes mostly specific to Firmicutes. This cluster co-evolves with *polC*, genes involved in cell division and genes involved in RNA degradation.

While already revealing, this first analysis cannot give us a detailed view of gene co-evolution as it is based on a considerably biased sample of genomes. Indeed, genomes have been chosen to be sequenced as a function of the history of biological studies in the academic world (and particularly oriented towards bacteria of medical interest), and not based on a view spanning the whole tree of life, with equal weight for all extant species. In general, finding the same frequency of gene occurrence in a clade does not imply that these genes are simultaneously present or absent in the same organisms. This is particularly true when clades are made of only a few organisms (Thermotogae, Acidobacteria, Chloroflexi, Deinococcus have less than twelve organisms). Naturally, this is much less so in clades that are well represented (Firmicutes and gamma-Proteobacteria comprize more than 250 organisms). Furthermore this preliminary analysis was only based on the essential genes of *B. subtilis*, and it is well established that functional ubiquity does not equate to gene ubiquity: a same function could be essential in other clades, but performed by a gene of a completely different descent.

In order to investigate more accurately gene co-evolution we needed therefore to analyze the occurrence profiles of all *B. subtilis *genes at the level of several phylogenetically distant model organisms rather than clades. To this aim, we designed a specific phylogenetic profile heuristics (PhyloProfile, see Methods) and based our exploration on the recently re-sequenced and re-annotated genome of *B. subtilis *[35] in parallel with that of the reference sequence and annotation of *E. coli *[36], an organism phylogenetically distant from *B. subtilis*. The method allowed us to identify clusters of genes that specificallly co-evolved with *dnaE *and *polC*.

### Genes co-evolving with dnaE

As a possibly ancestral protein, DnaE co-evolves with a very large number of genes. We used different co-evolution scores thresholds (0.50 to 0.90) to investigate how the proteins of the whole proteome clustered with DnaE. When the threshold was higher than 0.75, the number of genes co-evolving with DnaE stabilized to approximately 250 genes. As an example, using 0.77 as a threshold, we listed the genes co-evolving with DnaE from *B. subtilis *(Additional file 1). This list matched remarkably well with the key genes coding for the persistent functions that are required to allow reproduction and replication [37], substantiating the validity of the heuristics.

Most of the genes coding for the translation and transcription machineries, including factors and enzymes involved in modification and maturation of basic components of the machineries, were found in the list. This list also comprized the genes coding for the major metabolic pathways allowing construction of the cell: pyrimidine and purine biosynthesis and salvage, the core of the glycolytic/gluconeogenesis pathway (*eno, gapA, pgk, tpiA*...) and ATP synthase, as well as the secretion machinery. A number of components of the replication apparatus (DnaA, DnaC, DnaG, DnaN, GyrAB, LigA, PcrA, RnhB, SsbA) and genes involved in recombination and repair (Nth, Obg, PolA, RadA, RecA, RecG, RecN, RecO, RuvAB) were present as well in this set of proteins co-evolving with DnaE. Remarkably, a set of functions was missing from this list, that which corresponds to many components of RNA degradation (see below) [38]. Indeed, we found only a very limited set of RNA maturation and catabolism genes: *rnc *(coding for RNase III), *rnhB *(already mentioned, allowing degradation of RNA/DNA hybrid sequences during replication of the DNA lagging strand) and *pnpA*, a gene coding for an enzyme degrading RNA while preserving phosphate bond energy, polynucleotide phosphorylase.

Beside genes coding for known functions, a consistent set of genes coding for unknown functions was present in most bacterial clades. The corresponding list of the corresponding 33 « y » *B. subtilis *genes within the larger DnaE-related gene set is displayed in Table 1. These genes were further analyzed as they correspond to unknown or poorly identified functions that, if understood, would enhance considerably our understanding of bacteria. In the course of this exploration, we benefited from continuous re-annotation of the updated genome sequence [35]. Using the most recent publications, this limited the unknowns to at most 18 genes. Interestingly, the majority of the newly identified functions was involved in ribosomal RNA metabolism (*rsmI*(*yabC*), *rsmD(ylbH), rlmN(yloN), rimP(ylxS), hflX(ynbA), cpgA(yloQ)*), further substantiating the DnaE/translation connection. We also found in this set the hibernation promoting factor (*hpf(yvyD)*) and further genes involved in recombination and repair (*uup*(*yfmR*), *recD*(*yrrC*), *rarA*(*yrvN*)). All of these genes have a counterpart in *E. coli*. A gene, shorter in *B. subtilis *than in *E. coli*, codes for a function important for mRNA turnover, *rpsA *in *E. coli *and *ypfD *(renamed *rpfA*) in *B. subtilis*. This gene codes for ribosomal protein S1 in *E. coli *and there is a clear indication, in this case, that the ribosomal protein function has been superimposed on the general function of mRNA presentation to the degradosome [39]. This is a first indication that there is divergence between proteins functionally related to DnaE polymerase III in *E. coli *and *B. subtilis*, in particular at the level of RNA degradation.

**Table 1:** Genes of unknown function co-evolving with *Bacillus subtilis dnaE*

Label	B. subtilis	old name	E. coli	Function
BSU00200	yaaK	yaaK	ybaB	DNA binding protein^1^
BSU00360	**rsmI**	yabC	yraL	ribosomal RNA small subunit methyltransferase I^2^
BSU00390	yabD	yabD	ycfH	metal-dependent DNase^1^
BSU00480	yabJ	yabJ	yjgF	putative enzyme resulting in alteration of gene expression
BSU05910	ydiB	ydiB	yjeE	putative ATPase or kinase UPF0079
BSU05920	ydiC	ydiC	yeaZ	putative chaperone or protease
BSU05950	ydiF	ydiF	ybiT	putative ABC transporter (ATP-binding protein)^3^
BSU07370	**uup**	yfmR	ycbH	putative ABC protein involved in RecA-independent precise excision of transposons^1^
BSU07900	**rbn**	yfkH	yihY	putative ribonuclease BN^2^
BSU09240	yhcW	yhcW	yniC	putative phosphoglycolate phosphatase
BSU14350	yknX	yknX	ybjY (macA)	putative efflux permease^3^
BSU15010	**rsmD**	ylbH	yhhF	ribosomal RNA small subunit methyltransferase D^2^
BSU15380	ylmE	ylmE	yggS	conserved hypothetical protein
BSU15750	**rlmN**	yloN	yfgB	23S rRNA m2A2503 methyltransferase^2^
BSU15780	**cpgA**	yloQ	rsgA (yjeQ)	GTPase involved in ribosome and sacculus morphogenesis^2^
BSU16590	**rimP**	ylxS	yhbC	ribosome maturation factor^2^
BSU17430	**hflX**	ynbA	hflX	ribosome associating GTPase^2^
BSU21450	yolJ	yolJ	"-"	putative glycosyltransferase
BSU22880	**rpfA**	ypfD	"-" rpsA	RNA degradation presenting factor (ribosomal protein S1 homolog)^2^
BSU24790	yqgX	yqgX	ycbL	putative metal-binding hydrolase
BSU24890	**folN**	yqgN	ygfA	5-formyltetrahydrofolate cyclo-ligase
BSU25320	yqfG	yqfG	ybeY	putative metal-dependent hydrolase
BSU25620	yqeL	yqeL	ybeB	putative ribosomal maturation protein^2^
BSU27370	yrrL	yrrL	yceG	conserved hypothetical protein
BSU27390	yrrK	yrrK	yqgF	putative Holliday junction resolvase^1^
BSU27480	**recD**	yrrC	recD	exodeoxyribonuclease V alpha chain^1^
BSU27490	yrrB	yrrB	yciM	putative tetratricopeptide repeat family protein
BSU27530	**rarA**	yrvN	ycaJ	DNA-dependent ATPase^1^
BSU27700	yrbF	yrbF	yajC	component of the preprotein translocase
BSU28360	**rdgB**	ysnA	yggV (rdgB)	inosine/xanthosine triphosphate pyrophosphatase (subunit A)
BSU30680	ytjA	ytjA	yidD	conserved hypothetical protein
BSU35310	**hpf**	yvyD	yhbH	ribosome-associated sigma 54 modulation protein^2^
BSU36950	**tamT**	ywlC	yrdC	tRNA threonylcarbamoyladenosine biosynthesis protein^2^

1: DNA metabolism

2: RNA metabolism

3: transport

Starting with the *B. subtilis *nomenclature, we grouped the 18 remaining genes of unknown function according to common features, either extracted from the literature or using the neighborhood software STRING [40], with the hope to further uncover some of the associated functions. Despite its consistently valid outcomes we refrained from using our PhyloProfile to avoid circular validation. Interestingly, all but one (*yolJ*, a gene belonging to bacteriophage SPbeta, that disappeared from PhyloProfile when the threshold was increased) of these co-evolving genes have at least one clear ortholog in *E. coli*. *yaaK *belongs to an operon involved in DNA replication and repair, its STRING pattern further subtantiates this functional association. *yabD *and *yrrK yrrL *are connected together and relate to genes of the translation machinery (*metG, ksgA, serS, rsmG*) as well to genes involved in DNA metabolism and aromatic compounds biosynthesis. *yabJ *(*yjgF *in *E. coli*) codes for a putative enzyme with a 3D known structure conserved in all three domains of life [41]. Here, it is related to intermediary metabolism via aminotransferase PatB, the control of purine metabolism (*purR, relA, gmk, rdgB*), isoleucine biosynthesis and to proteolysis (via ClpX). In contrast, in *E. coli*, it is related to catabolism of threonine, pyrimidine metabolism and to a network of genes of unknown function. *ydiB *(coding for a putative shikimate dehydrogenase [42]), *ydiC *and *ydiF *connected to ribosome maturation and sulfur metabolism. *yhcW *connects to *ilvA *and translation; *yknX*, in an operon coding for a putative efflux permease, down regulated in the presence of benzoate [43]; *ylmE *to cell division and proline and purine/polyphosphate metabolism; *yqgX *to translation via aspartyl and histidyl tRNA synthetases, as well as D-tyrosine deacylase, transformylase and RelA; *yqfG *to translation and phosphate metabolism; *yqeL *and *ytjA *to translation and tRNA modification; finally, *yrrB *and *yrbF *are connected to several genes involved in tRNA modification.

In summary, DnaE co-evolves with most of the replication, recombination, translation, transcription and secretion machineries, and with some of the core metabolic biosynthetic pathways. We noted however the absence of a consistent RNA degradation pathway as well as most enzymes of the envelope biosynthetic pathways. This suggested that both RNA degradation and envelope biosynthesis, while functionally essential, might derive from different descent in different bacterial clades.

### Genes co-evolving with polC

The list of the 162 genes co-evolving with *polC *differed considerably from that co-evolving with *dnaE *(Additional file 1). In particular, among those, 69 were genes of unknown function, a considerable proportion (Table 2). Interestingly, when analyzing their neighborhoods with STRING, we observed that there was seldom any connection with the *dnaE*-related networks, as if *polC *had evolved from a completely different origin. We also noticed that almost all genes in the list, while present in *B. subtilis*, do not have an ortholog in *E. coli*. This is exactly the opposite of what we found with genes co-evolving with *dnaE*. As for DnaE, the PolC-related proteins could be clustered into functionally significant groups (Tables 2 and 3). Remarkably, many of these clusters correspond to some aspect of RNA metabolism. Using STRING, these genes could be further clustered into seven groups (Table 3), the other ones remaining isolated.

**Table 2:** Genes of unknown function co-evolving with *Bacillus subtilis polC*

label	B. subtilis	E. coli	Function	Functional connection	String groups
BSU00030	yaaA	ybcJ	putative RNase or phosphorylase; conserved in yeast; very weak in *E. coli*^2^	RNA/DNA metabolism	**group 1**
BSU00330	yabA dnaH	"**_**	subunit of the DNA replication complex^1^	DNA polymerase and "y" network	**group 1**
BSU00340	yabB	"**_**	putative RNA methyltransferase^2^	RNA metabolism	**group 1**
BSU00630	yabR	"**_**	putative RNA degradation protein; polyribonucleotide nucleotidyltransferase or phosphorylase^2^	degradosome network with RfaA	group 2
BSU31390	yugI	"**_**	putative RNA degradation protein or phosphorylase or nucleotidyl transferase;	degradosome network with RfaA	group 2
BSU01750	ybbP	"**_**	homolog of YabR and RpfA^2 ^DAC domain protein present in Archaea		**group 3**
BSU01760	ybbR	"**_**	substrate for Sfp phosphopantetheinyl transferase-catalyzed protein labeling by small molecule-CoA conjugates		**group 3**
BSU40510	yybT	".	phosphodiesterase acting on cyclic dinucleotides; possibly nanornase^2^	RNA metabolism	**group 3**
BSU15060	ylbM	"**_**	conserved hypothetical protein, found in a ribonuleoprotein complex in Mus musculus^2^	RNA metabolism	**group 3**
BSU25630	yqeK	"**_**	putative hydrolase		**group 3**
BSU25680	yqeG	".	putative hydrolase		**group 3**
BSU11030	yitL	"**_**	RNA-binding protein^2^	RNA metabolism	**group 3**
BSU19680	yozE	".	conserved hypothetical protein^2^	RNA metabolism	**group 3**
BSU24860	yqgQ	"**_**	putative single strand nucleic acid binding protein^2^	RNA metabolism	**group 3**
BSU14540	ykzG	"**_**	omega subunit of RNA polymerase^2^	RNA metabolism	**group 3**
BSU16610	ylxR	"**_**	putative RNA binding protein; putative new fold^2^	RNA binding	**group 3**
BSU27380	yrzB	"**_**	putative anti-sigma factor		**group 3**
BSU15830	yloU	"**_**	conserved hypothetical protein		**group 3**
BSU15840	yloV	dhaL	putative dihydroxyacetone/glyceraldehyde kinase		**group 3**
BSU08680	ygaC	"**_**	putative factor, domain associated with ribonuclease E and G, possibly involved in Fe-S group formation^2^	degradosome network	**group 3**
BSU15970	ylxM	"-	conserved hypothetical protein^2^	RNA/DNA metabolism	**group 3**
BSU27950	ysxB	"-	conserved hypothetical protein with ribosomal function^2^	ribosome	**group 3**
BSU16000	ylqC	"-	putative RNA binding protein^2^	RNA binding	**group 3**
BSU25670	yqeH	"-	GTPase involved in ribosome 30S assembly^2^	ribosome	**group 3**
BSU16970	ymdB	"-	putative phosphoesterase		**group 3**
BSU27400	yrzL	"-	conserved hypothetical protein functionally linked to alanine tRNA loading^2^	RNA metabolism	**group 3**
BSU32310	yutD	"-	conserved hypothetical protein^2^	RNA metabolism	**group 3**
BSU14640	yktA	"-	conserved hypothetical protein		**group 3**
BSU15000	ylbG	"-	conserved hypothetical protein		**group 3**
BSU17880	ynzC	"-	conserved hypothetical protein		**group 3**
BSU22190	ypsA	"-	conserved hypothetical protein		**group 3**
BSU07420	yfmM	"-	putative polyphosphate-AMP phosphotransferase		**group 3**
BSU07430	yfmL	"-	putative ATP-dependent RNA helicase^2^	RNA metabolism	**group 3**
BSU00970	yacP	"-	putative ribonuclease with PIN and NYN domains; similar to eukaryotic RNases^2^	RNA metabolism	group 4
BSU23950	yqjA	"-	conserved hypothetical protein putative		group 4
BSU32280	yutG	"-	phosphatidyl-glycerophosphatas e A		group 4
BSU16845	ymfF	"-	putative metalloprotease	protein metabolism	group 4
BSU16860	ymfH	"-	putative processing protease	protein metabolism	group 4
BSU09800	yheA	"-	conserved hypothetical protein		group 4
BSU09930	yhaM	"-	3'-5' exonuclease^2^	DNA/RNA metabolism	group 4
BSU01450	ybxA ecfA	"-	energizing coupling factor ABC multiple influx transporter (ATP-binding protein)^3^	specific transport	**group 5**
BSU01460	ybaE ecfB	"-	energizing coupling factor ABC multiple influx transporter (ATP-binding protein)^3^	specific transport	**group 5**
BSU01470	ybaF ecfT	"-	permease component of the EcfAB influx transporters^3^	specific transport	**group 5**
BSU11590	yjbL	"-	putative phosphatase		group 6
BSU11600	yjbM	"-	(p)ppGpp synthetase		group 6
BSU11620	yjbO	rluD	pseudouridylate synthase^2^	RNA metabolism	group 6
BSU29820	ytpR	"-	putative protein with RNA binding domain^2^	RNA binding	**group 7**
BSU29840	ytpP	"-	putative thiol-disulfide oxidoreductase with thioredoxin domain^5^	sulfur metabolism	**group 7**
BSU29860	ytoP	frvX	glutamyl aminopeptidase; deblocking enzyme (wrong annotation in *E. coli*)	protein metabolism	**group 7**
BSU13480	ykrK	"**_**	conserved hypothetical protein		
BSU14100	ykuJ	"**_**	putative RNA-specific modification enzyme subunit^2^	RNA metabolism network of "y" genes	
BSU14130	ykuL	"**_**	conserved hypothetical protein		
BSU15410	ylmH	"**_**	factor involved in shape determination, RNA-binding fold^2^		
BSU17440	ynbB	"**_**	putative C-S lyase^5^	sulfur metabolism	
BSU17699	ynzK	"**_**	putative membrane protein		
BSU17910	yneF	"**_**	conserved hypothetical protein; methionine-rich^5^	sulfur metabolism	
BSU19620	yodJ	"**_**	D-alanyl-D-alanine carboxypeptidase lipoprotein^4^		
BSU24260	yqxC	"**_**	putative methyltransferase with RNA binding domain^2^	RNA binding	
BSU28650	ysgA	"**_**	putative RNA methylase^2^		
BSU29780	ytxG	pqiB	homolog involved in DNA repair in *Mus musculus*^1^	DNA repair	
BSU29980	ytjP	"**_**	putative dipeptidase	protein metabolism	
BSU30460	ytrA	"**_**	transcriptional regulator (GntR family)		
BSU30490	ytqB	"**_**	putative RNA methylase^2^	RNA metabolism	
BSU30870	ytcB	"**_**	putative UDP-glucose epimerase^4^		
BSU36340	ywpE	"**_**	putative sortase		
BSU36910	ywlG	"**_**	conserved hypothetical protein; present in Archaea and Eukarya		
BSU37340	ywiB	"**_**	putative RNA binding protein possibly involved in aminoacyl-tRNA editing^2^	RNA binding	
BSU38499	ywzH	"**_**	conserved hypothetical protein		
BSU40939	yyzM	"**_**	conserved hypothetical protein		

1: DNA metabolism

2: RNA metabolism

3: transport

4: envelope metabolism

5: sulfur metabolism

**Table 3:** Genes groups identified using the STRING software [40]

Groups	STRING genes
group 1	yazA yabB yaaT yaaR yabC yabA yaaA yadB tmk holB dnaN dnaA dnaD recF
group 2	yabR rpfA yugI yabMNOPQ hprT rpmE divIC rpfA(ypfD) alaRT ftsZ gatA ppiB acpS alaS aroD cbsA cca cotD csbA ctsR cysS defB dltC dltD dnaB dnaC dnaD ecsB ffh ftsK ftsL ftsY glcU glmM gluKP glyQS gpsB hcrA infB jag kapB lepA ltaSA lysA mecAB mtnN murB mutSB nadD nrnA nusA obg polC priA prmA rbfA recA recG recU recX rex ribC ribT rimM rmpA rnc rnhB rnjA rplI rplS rplU rpmB rpmF rpoZ rpsP rpsR rsiW
group 3	scpAB sdaAA sdaAB sfp sigW smc speA spoIIIAD spoIVFA spoIVFB spoVS suhB sul tkt trmD truB ung uppS yaaQ yaaR yabF yabO yazC ybbP ybbR ydcK yebG yerH yetN yfhJ yfmKLMO ygaBCD yhaL yhcV yheA yisL yitKL yizA yktAB ykzG ylaH ylbFGMNP yloUV ylqC ylxMPQRS ymdAB yneABER ynzC yozE ypeQ ypsA yqeGHIKLM yqfC yqgNQY yqhP yqzF yrdA yrrKLM yrzBL yslB ysxB ytpI ytwI yuiB yutDEM yuzB yvcKL ywfO ywnH yybPRST yycHI
group 4	yacOP yueI yazC yvcS ywhD ylaL yozC yetH yqjB artMQ sigH ispDF cysS gltX cysE radA yqjAB mecB yutGH yneR ftsL cinA veg ydaS ypjQ yheA yojF ymxH fabR ymfFGHIJ lgt mreC recFG ctsR ybaF yheAB yqgQ yhaMO ywbD aroCF tyrA cwlJ yhaMNO rpe tmk rsgA ksgA thiN smpB topA cca
group 5	ksgA ecfAB ecfT ytlC cbiOA cbiOB rpsI rpoA truA rplQ rplM skfE ykoCD yuiG bioY
group 6	ywpF ywhD ymfK ywzC ylaL ywjG ypjB bofC ppnKA ndk rpoZ rpoABCE relA pyk folE yjbLMO yaaC ytzF lspA prfB truAB rluB
group 7	yrvN polA msrB pheST metG alaS ispG ytpPQRST ytoPQ ysdC ytzB comEA

Group 1 clusters a subunit of the DNA replication complex, DnaH(YabA), together with putative RNA binding proteins (YaaA and YabB). Distant homologs exist outside of Firmicutes, in particular in Eukarya.

Group 2 comprizes proteins involved in the Firmicute degradosome network and possibly involved in stress management (YabR and YugI (general stress protein GSP13 [44], essential in *Staphylococcus aureus *[45]), both displaying a S1 motif [46]).

Group 3, the largest group, is organized around several proteins, most of which small or very small. They have often a known 3D structure but do not yet have an idenfied function. They are involved in RNA metabolism (transcription, RNA modification and turnover): YitL, an RNA binding protein, RnpZA(YkzG), a component of the omega subunit of RNA polymerase, YktA (possibly involved in polyamine metabolism, YlbG (possibly involved in activity of a conserved small RNA, CsfG [47]), YozE, of unknown function, YqgQ (putative single-strand nucleic acid binding protein involved in transcription [48]), and finally YrzL (essential in *S. aureus *[45]) and YutD, unknown proteins that are possibly hydrolases. With STRING we observed that this cluster is further connected to RnjA, the non-orthologous functional equivalent of RNase E in proteobacteria [49]. Many of these proteins are members of UniProt Unknown Protein Families (UPFs), most of which associated to Firmicutes and sometimes Archaea and Eukarya. This cluster further connects via YqgQ to DNA replication and recombination, to energy-dependent proteases, to divalent metal transporters and to proteins involved in shaping the cell. Via YloU (with a paralog, YqhY), YloV (dihydroxyacetone kinase-like), YlxM (conserved in *Mycoplasma *sp.), YnzC, YfmM (proposed to code for polyphosphate-AMP phosphotransferase in *Staphylococcus epidermidis *[50]), YmdB (putative phosphoesterase), YqeG (essential in *S. aureus *[45]), this cluster again comprises a large number of UPFs. It has some connection with the recombination machinery (RecA, RecG) and the envelope (PlsX, Ffh). YloU has similarity to yeast S-adenosylmethionine-dependent tRNA (uracil-5-)-methyltransferase. This may be significant, because in A+T-rich Firmicutes this activity differs from that in most other organisms, TrmFO, the methylating activity, depending on methylene-tetrahydrofolate rather than AdoMet, thus suggesting recruitment for another RNA modification activity [51]. Finally a sub-cluster of this large group associates YlbM (member of a ribonucleoprotein complex), YqeG (hydrolase), YqeK, YqeH (phosphohydrolase), and YybT (a putative phosphodiesterase, with motif GGDQV related to that of cyclic-diGMP synthesis and degradation). It is connected to Cca, RplI and YqeI, a putative RNA binding protein. Again, this cluster has a clear RNA metabolism flavour.

Group 4 comprises YmfF and YmfG, two peptidyl hydrolases of unknown substrates, perhaps associated to transport of peptidyl siderophores [52] and connects to YheA (related to metabolism of aromatics (YwbD, AroC, TyrA, AroF)). It contains proteins such as YqjA (a putative membrane bound protein), YacP (possibly involved in ribosomal RNA maturation), connected to a network with tRNA synthetases CysS and GltX and with sigma factor SigH. It also comprises YhaM (a 3'-5' oligonucleotidase) and YutG (a putative phosphatidyl-glycerophosphatase A).

Group 5 has now an identified function: it corresponds to the EcfABT energy-dependent activating part of a multi-substrate transporter [53].

Group 6 clusters together YjbL, a putative phosphatase, YjbO, similar to RluD pseudouridylate synthase and YjbM, connected to RelA and RNA polymerase (RpoABCZ) as expected from its putative function in ppGpp synthesis and turnover. This homolog of RelA/SpoT seems to be specific to A+T-rich Firmicutes. It is also connected to riboflavin and folate biosynthesis.

Group 7 associates YtpP and YtpR, connected to tRNA synthetases PheST, AlaS and MetG, as well as DNA polymerase PolA and methionine sulfoxide reductase MsrA. It contains also YtoP (FrvX, a glutamyl aminopeptidase in *E. coli*).

Finally, the 20 remaining genes do not belong to clear clusters and do not have obvious functions. Yet, six among them (*ykuJ, ylmH, yqxC, ysgA, ytqB, ywlB*) code for proteins having RNA binding properties. *ynbB *and *yneF *are connected to sulfur metabolism and possibly tRNA modification. Protein YtxG has an homolog involved in DNA repair in *Mus musculus. *YodJ and YtcB are protein likely to be involved in murein synthesis or turnover.

## Discussion

Cells encode several DNA polymerases that fulfill a variety of reactions: genome replication, repair and recombination. Eukarya have two types of DNA replicases. In the same way, the genomes of the Firmicutes clade have a unique heterodimeric DNA polymerase III α-subunits, PolC and DnaE [54]. In contrast the *E. coli *replicase is made of two identical DnaE subunits. The difference between the replicase of *E. coli *and that of *B. subtilis *is further reflected by the composition of the other subunits of DNA polymerase III that differ from those in other clades. In *E. coli*, a single protein, the AAA+ ATPase DnaC (counterpart of DnaI in *B. subtilis*), is used to load helicase DnaB [55]. In contrast, in *B. subtilis *and other low-G+C Firmicutes, three different proteins, DnaD, DnaB (no counterpart in E. coli, used for loading to DnaC), and the AAA+ ATPase DnaI, are needed to load the replicative helicase (DnaC in B. subtilis is the counterpart of DnaB in E. coli). DnaA binds first, followed by DnaD and then DnaB, and finally the DnaI-mediated loading of helicase occurs [56].

The existence of two replicases implies a physical difference in the replication process that should be reflected in a bias in nucleotide strand composition. Analysis of compositional strand asymmetries of prokaryotic genome sequences in terms of the presence or absence of PolC has found not only a correlation with PolC [12], but also with a purine asymmetry [7]. This latter asymmetry is however probably not the result of the physical differences in the leading and lagging strands replication, but, rather, the consequence of the increase in the gene content of the leading strand in organisms with PolC, which alleviates conflicts between the transcription and replication machineries [10,57]. In particular it was noticed that G seems to favour the leading strand in most bacterial genomes, which fits with an over representation of genes in this strand in Firmicutes, as coding regions are overrepresenting GNN codons [58].

DNA composition analysis is of limited interest per se, as it does not provide much information about gene functions. The present study showed that while the functional connection of the DnaE subunit spans the whole domain Bacteria, with almost all functions that are expected to make a minimal genome, this is not so of the PolC co-evolving genes. Furthermore, the latter group of genes seems highly specific and related to original features of biological processes, RNA metabolism in particular.

PolC is the subunit of DNA replicase that replicates the leading strand of the DNA double helix in Firmicutes. Structural data on the PolC family of replicases shows that it evolved separately from DnaE [59]. This process requires in addition to PolC, ten other proteins. In addition to these 11 proteins, lagging strand replication requires DnaE and primase [56]. Progression of the replisome implies that DnaE uses Okasaki fragments as primers, and these fragments need to be removed, so that they do not interfere with progression of PolC-mediated leading strand replication progression [60]. It is even suspected that, because replication of the lagging strand must be slow, bacterial replicases are made of not of two, but three replication subunits, two of them involved in replication of the lagging strand [61].

In this context, management of the degradation of RNA derived from Okazaki fragments has a central importance. In particular degradation of nanoRNAs, fragments of size smaller than 5 nts, becomes crucial, as they can enter the replication bubble. It is established that PolC discriminates against RNA primers while DnaE uses RNA primers efficiently [54,56]. As a consequence it can be expected that PolC co-evolved with a particular type of RNA degradosome. Indeed, in the present work, many proteins co-evolving with PolC could be seen as associated to properties of a Firmicutes-specific degradosome (or downstream from it) [39], comprizing nanoRNases such as NrnA [62], an RNase which degrades short oligoribonucleotides (and which is present in the smallest genome of an autonomous living organism, *M. genitalium*) but also RnmV, MrnC(YazC), YacP, Rny(YmdA), YhaM, YkzG, YlbM, YlmH, RnhC(YsgB) and YybT, proteins that are not present in the *E. coli *degradosome (Additional file 1). The presence of a particular type of RNase H (RnhC), required to hydrolyze RNAs belonging to RNA-DNA hybrids, essential in *B. subtilis *[63], is particularly revealing, as it further supports the conjecture that the co-evolution we observed is related to RNA turnover. A further related observation is that there is no global counterpart of the degradosome proteins in those evolving with DnaE, except for the core phosphorylase, polynucleotide phosphorylase, and a specific RNase H (RnhB) of descent different from that of RnhC. NanoRNases (essential for degrading Okazaki fragments) have now been identified from three descents: Orn in gamma-Proteobacteria, NrnA in Firmicutes) and NrnC in alpha-Proteobacteria [64]. This indicates that there may exist more than two degradosomes in the Bacteria domain. Further work similar to the one presented here will tell.

Finally, we remarked that the genes that co-evolve with *polC *have often counterparts in the three domains of life. This suggested that, despite their absence from the genes co-evolving with the majority of the essential cellular processes, these genes are of very ancient descent. Interestingly, many of the corresponding functions are related to RNA metabolism but also to phosphate or polyphosphate metabolism.

## Conclusion

Analysis of the genes co-evolving with the two forms of DNA replicase found in Bacteria, DnaE and PolC revealed that, while DnaE co-evolved with the translation and transcription machineries, PolC co-evolved with proteins that do not belong to the same group. In particular PolC co-evolved with a form of the RNA degradation machinery that is distinct from that characterized in gamma-Proteobacteria, the *E. coli *degradosome [38]. Among other possibilities, this observation suggests that, while there may exist a last common ancestor to the translation/transcription machinery, this was probably not so for the machineries leading to RNA turnover, which may have appeared independently on several occasions. The RNA world, that developed RNA-centered metabolism [65], predated the RNA genome world, in which RNA synthesis and turnover must have been essential. Our observations are consistent with the discovery of DNA and DNA replication at least twice [66] suggesting that the origin of present living organisms was a community of organisms developing more or less independently from one another (the progenote hypothesis [67,68]), splitting and fusing as time elapsed until the present domains were more stably defined.

## Authors' contributions

SE and DV designed and implemented the PhyloProfile algorithm. SE wrote part of the manuscript. CM coordinated the implementation in the MicroScope platform. AD designed and coordinated this study, performed the biological interpretation of the results and wrote the bulk of the manuscript. All authors read, modified and approved the final manuscript.

## Supplementary Material

Additional file 1 **Gene clusters obtained using PhyloProfile with a threshold of 0.77**. The first two clusters comprise the *dnaE *and the *polC *clusters. At the end of the list many clusters contain only two genes that co-evolve significantly. Click here for file
